# Clinical monitoring of cardiac output assessed by transoesophageal echocardiography in anaesthetised dogs: a comparison with the thermodilution technique

**DOI:** 10.1186/s12917-017-1227-9

**Published:** 2017-11-09

**Authors:** Matheus M. Mantovani, Denise T. Fantoni, André M. Gimenes, Jacqueline R. de Castro, Patrícia B. Flor, Keila K. Ida, Denise S. Schwartz

**Affiliations:** 1 Departamento de Clínica Médica, Faculdade de Medicina Veterinária e Zootecnia, Universidade de São Paulo, São Paulo, Brazil; 2 Departamento de Cirurgia, Faculdade de Medicina Veterinária e Zootecnia, Universidade de São Paulo, São Paulo, Brazil; 3 Laboratório de Investigação Médica 8, Anestesiologia, Faculdade de Medicina, Universidade de São Paulo, São Paulo, Brazil; 4 Faculdade de Medicina Veterinária e Zootecnia da Universidade Federal de São Paulo, Av. Prof Dr Orlando Marques de Paiva 87, São Paulo, SP 05508-270 Brazil

**Keywords:** Swan Ganz, Transgastric, Doppler, Aortic flow, Minimally invasive

## Abstract

**Background:**

Cardiac output (CO) is an important haemodynamic parameter to monitor in patients during surgery. However, the majority of the techniques for measuring CO have a limited application in veterinary practice due to their invasive approach and associated complexity and risks. Transoesophageal echocardiography (TEE) is a technique used to monitor cardiac function in human patients during surgical procedures and allows CO to be measured non-invasively. This prospective clinical study aimed to compare the transoesophageal echocardiography using a transgastric view of the left ventricular outflow tract (LVOT) and the thermodilution (TD) technique for the assessment of CO during mean arterial pressure of 65–80 mmHg (normotension) and <65 mmHg (hypotension) in dogs undergoing elective surgery. Eight dogs were pre-medicated with acepromazine (0.05 mg/kg, IM), tramadol (4 mg/kg, IM) and atropine (0.03 mg/kg, IM), followed by anaesthetic induction with propofol (3–5 mg/kg IV) and maintenance with isoflurane associated with a continuous infusion rate of fentanyl (bolus of 3 μg/kg followed by 0.3 μg/kg/min). The CO was measured by TEE (CO_TEE_) and TD (CO_TD_) at the end of expiration during normotension and hypotension (induced by isoflurane).

**Results:**

There was a strong positive correlation between CO_TEE_ and CO_TD_ ​​(*r* = 0.925; *P* < 0.0001). The bias between CO_TD_ and CO_TEE_ was 0.14 ± 0.29 L/min (limits of agreement, −0.44 to 0.72 L/min). The percentage error of CO measured by the two methods was 12.32%. In addition, a strong positive correlation was found between CO_TEE_ and CO_TD_ during normotension (*r* = 0.995; *P* < 0.0001) and hypotension (*r* = 0.78; *P* = 0.0223).

**Conclusions:**

The results of this study indicated that the transgastric view of the LVOT by TEE was a minimally invasive alternative to clinically monitoring CO in dogs during anaesthesia. However, during hypotension, the CO obtained by TEE was less reliable, although still acceptable.

## Background

Cardiac output (CO) is an important haemodynamic parameter to monitor in patients during surgery. However, the majority of the techniques for measuring CO have a limited application in veterinary practice due to their invasive approach and associated complexity and risks [1].

The thermodilution technique (TD) is the gold standard for monitoring CO in veterinary medicine [2, 3]. It requires the insertion of a catheter into the pulmonary artery through the jugular vein, and the application of a cold solution to calculate the CO by temperature differences between the blood within the right atrium and the pulmonary artery [4]. Therefore, the TD technique is an invasive and sometimes limited method considering the risks associated with the development of arrhythmias, infection, thrombosis, and rupture of the pulmonary artery [5, 6].

Transoesophageal echocardiography (TEE) is a technique used to monitor cardiac function in human patients during surgical procedures [7, 8]. The Doppler technique allows CO to be measured non-invasively by multiplying the velocity-time integral (VTI) of blood flow through the transversal area of the vessel (CSA) with heart rate (HR) (CO = CSA x VTI × HR) [9].

A transgastric approach on TEE proposed for visualisation of the left ventricular outflow tract (LVOT), which has been allowed to quantify the aortic flow and, therefore, to calculate CO in human patients during surgery, has shown a good correlation with TD [7, 8, 10]. Although TEE is used for interventional procedures in veterinary patients [11, 12], no studies have described the transgastric approach of LVOT, the CO measured through this view, or the accuracy of the technique.

Therefore, the aim of this study was to compare the TEE transgastric view of the LVOT and the TD for measuring CO in anaesthetised dogs during surgery. The hypothesis is that CO can be accurately and non-invasively monitored using the TEE technique during clinical anaesthesia in dogs.

## Methods

### Animals

The study was authorized by written consent of the owners and the protocol (1936/2012) was approved by the university’s ethical committee in the use of animals (CEUA). Eight dogs ASA 1 or 2, weighing 24.5 ± 2.1 kg, and undergoing surgery in the Veterinary Hospital of our institution were included in the study. The sample size for paired data was calculated using power analysis, indicating that a minimum of 8 dogs per group was required to have a 80% chance (with 5% risk) of detecting a difference of 0.78 L/min in the CO between groups and considering a standard deviation of 0.8 L/min.

### Anaesthesia and monitoring

Food was withheld for 12 h and water for 4 h before anaesthesia. All animals were pre-medicated with acepromazine (0.05 mg/kg, IM; acepromazina, Syntec, Brazil), tramadol (4 mg/kg, IM; Tramadon, Cristália, São Paulo, Brazil), and atropine (0.03 mg/kg, IM; Plasmodex, Isofarma, Brazil). After 15 min, the cephalic vein was catheterised, and anaesthesia was induced with propofol to effect (3–5 mg/kg; Propovan, Cristália), administered through the catheter. After orotracheal intubation, anaesthesia was maintained with an initial end-tidal isoflurane concentration (ET_ISO_) of 1.3% in 70% oxygen associated with fentanyl (bolus of 3 μg/kg followed by a constant rate infusion of 0.3 μg/kg/min; Fentanest, Cristália).

Neuromuscular blockade was instituted by rocuronium (0.6 mg/kg, IV; Esmeron, Organon, São Paulo, Brazil), and the mechanical ventilation (rebreathing circuit with a microprocessor-controlled anaesthesia ventilator; Shogun, Takaoka, São Paulo, Brazil) was then started using the pressure-controlled ventilation mode, with a peak inspiratory pressure of 10 cmH_2_O, inspiratory-to-expiratory time ratio of 1:2, and a respiratory rate (RR) adjusted to maintain an end-tidal carbon dioxide concentration (ETCO_2_) of 35–45 mmHg.

A 7F pulmonary artery catheter (Swan Ganz; Edwards Lifesciences, Irvine, CA, USA) was aseptically and percutaneously introduced into the right jugular vein using the Seldinger technique in dogs in dorsal recumbency. The distal sensor of the catheter was located in the pulmonary artery, which was confirmed by waveform analysis (DX2020; Dixtal, Brazil) and by transoesophageal echocardiographic imaging. Pressure transducers were connected to a multi-parametric data collection system for continuous monitoring of the pressures and waveforms (DX2020; Dixtal).

Heart rate (HR), RR and pulse oximetry (SpO_2_) were monitored using a multiparametric monitor (DX2020; Dixtal). The end-tidal isoflurane concentration (ET_ISO_) and ETCO_2_ were assessed by using a gas analyser (PoetIQ2-8500Q; Criticare Systems, WI, USA). The invasive systolic, mean and diastolic pressures (SAP, MAP and DAP, respectively) were monitored by catheterisation of the dorsal podal artery. All cardiopulmonary data were monitored continuously and registered at the same time-points as the CO measurement. A continuous infusion of lactated Ringer’s solution was delivered throughout (5 mL/kg/h).

### Cardiac output measuring

The cardiac output assessed through the thermodilution technique (CO_TD_) was measured at the end of expiration by administering 3 mL of 5% glucose at 5°C into the right atrium through the pulmonary artery catheter. The temperature change was detected by the thermosensitive tip of the catheter located into the pulmonary artery, and the CO value was automatically calculated by the multiparametric monitor (DX 2020; Dixtal). The mean value of three measurements within ±10% was registered as CO_TD_.

**Fig. 1:**
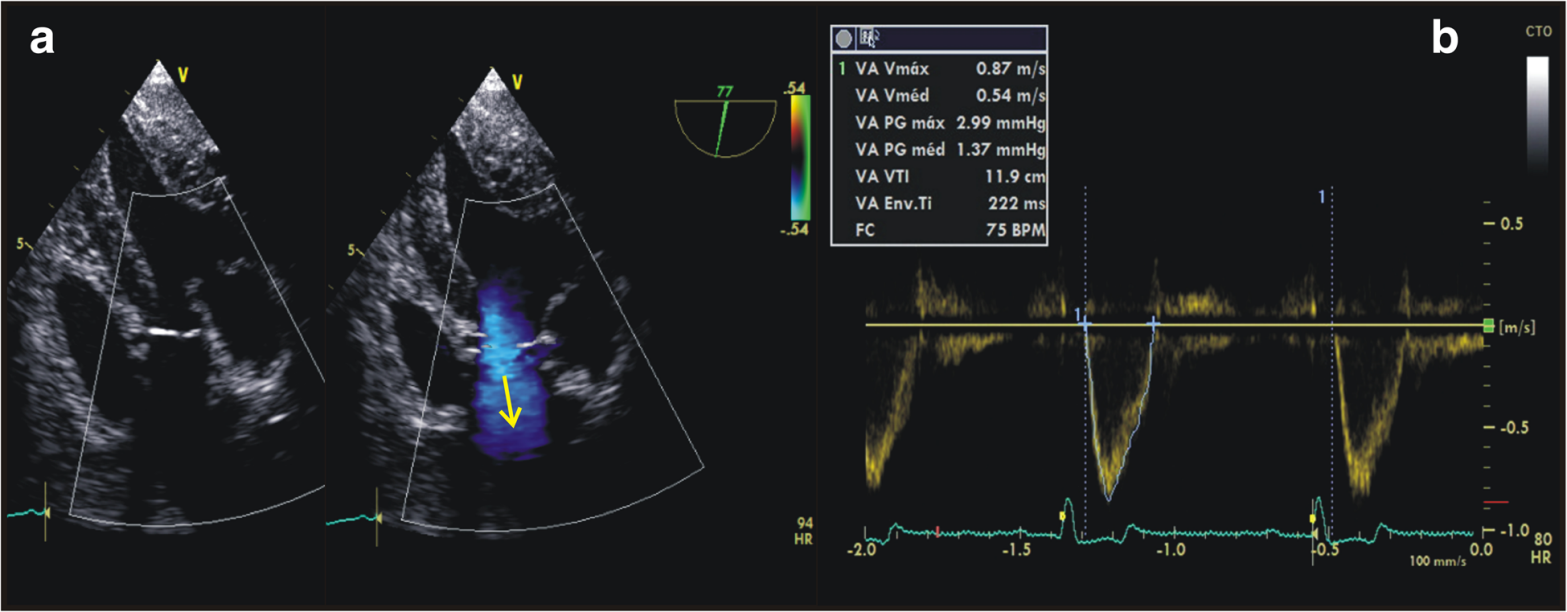
Transgastric view of the left ventricular outflow tract assessed by transoesophageal echocardiography. **a** The almost parallel alignment between the volume sampled by the pulsate Doppler and the aortic blood flow (arrow) was obtained through this view. **b** The velocity-time integral (VTI) of aortic flow was calculated by the outline of the aortic blood flow

The cardiac output assessed through transoesophageal echocardiography (CO_TEE_) was determined by using a multiplanar 2–5 MHz transoesophageal ultrasound transducer introduced into the mouth and passed into the oesophagus until visualisation of the aortic valve through the transverse caudal view. At this window, the planimetry of the aortic valve was performed to measure the transverse area of the aorta, as previously described by Boon [9]. Then, the transducer was inserted into the gastric cavity for visualisation of the short axis of the left ventricle at the level of the papillary muscles through the transverse transgastric view by ventroflexion movements of the transducer. At this level, the LVOT and the ascending portion of the aorta could be visualised by rotation of the ultrasonographic beam to approximately 90°-120° (Fig. 1a), allowing for a better alignment between the volume sampled by the pulsate Doppler and the aortic blood flow. The transgastric view of the LVOT could be improved by slight adjustments in the position of the transducer, which included the rotation or ventroflexion movements. From the transgastric view of the LVOT, the aortic blood flow was obtained via pulsate Doppler with the volume sampled immediately below the aortic valve. The velocity-time integral of aortic flow (VTI_aortic_) was calculated by the outline of the aortic blood flow (Fig. 1b). The CO_TEE_ corresponded to the mean of three consecutive values calculated by the formula CO_TEE_ = CSA × VTI_aortic_ × HR at three consecutive cardiac cycles, respectively.

The CO_TD_ and CO_TEE_ were always measured at the same time at expiration, and the values of CO_TD_ were not revealed to the echocardiographic examiner. Both techniques were applied after 20 min of anaesthetic stabilisation at a MAP of 65–80 mmHg (normotension) and then at an MAP <65 mmHg (hypotension) induced by increasing the ET_ISO_ [18]. Arterial hypotension was treated immediately after measuring CO by reducing ET_ISO_ to baseline values. If necessary, hypotension was treated by further decreasing ET_ISO_ and by administering a bolus of crystalloids or colloids, and a bolus of 0.1 mg/kg of ephedrine until normalisation of MAP; these treatments, if any, were registered accordingly.

### Statistical analysis

The distribution of all variables was analysed for normality using the Shapiro-Wilk test. The variables with a normal distribution were analysed using the paired Student t-test. The variables with a non-parametric distribution were analysed using the Wilcoxon matched-pairs signed rank test.

The Pearson coefficient, the intraclass correlation coefficient (ICC), and a model of linear regression (CO_TEE_ as the dependent variable and CO_TD_ as the independent variable) were calculated to assess the association between CO_TEE_ and CO_TD_.

The agreement between CO_TD_ and CO_TEE_ was assessed by the Bland-Altman test [16]. The bias between the techniques was calculated as the mean of the differences between the CO measured by each technique. The limits of 95% agreement were calculated as the bias ± (2 × standard deviation). The ratio of the agreement limits for the CO value (percentage of the error; expressed as %) was calculated as 100 × (± 2 × standard deviation) ÷ CO (resulting from the overall mean of the two methods). A *P* -value <0.05 was considered statistically significant for all analysis.

Repeatability CO_TEE_ measurements was assessed using five animals. Each echocardiogram was evaluated three times in a given day by the same observer (intraobserver analysis) and by another independent observer (interobserver analysis). The VTI_aortic_ and CSA were measured three times in consecutive cardiac cycles in the same frame. The resulting mean values and standard deviations were used to determine the coefficients of variation. The echocardiogram analyses were performed offline.

## Results

Animals included in the study are shown in Table 1. The haemodynamic and respiratory variables, and the CO of each animal included in the study are presented in Tables 2 and 3, respectively. The CO, SAP, MAP, and DAP had a normal distribution and were analysed using paired t-Student test. The HR, RR, ETCO_2_, ET_ISO_, and SpO_2_ had a non-parametric distribution and were analysed using the Wilcoxon test.

**Table 1:** Breed, genre, bodyweight, surgery and ASA classification of the anaesthetic risk of 8 dogs undergoing cardiac output monitoring assessed by the transesophageal echocardiography and thermodilution techniques

Animal	Breed	Gender	Bodyweight (kg)	Surgery	ASA
1	Mixed-breed	F	17.2	Orthopaedic procedure	2
2	Mixed-breed	F	16.8	Laparotomy	2
3	Mixed-breed	M	24.3	Orthopaedic procedure	2
4	Boxer	M	29.5	Laparotomy	2
5	Pit bull	F	23	Orthopaedic procedure	2
6	Mixed-breed	F	26	Orthopaedic procedure	2
7	Mixed-breed	F	22.6	Laparotomy	2
8	Beagle	M	18.4	Ophthalmic procedure	1

ASA: *American Society of Anaeshesiologists* classification of physical status

**Table 2:** Cardiorespiratory parameters assessed in 8 anaesthetised dogs during surgery

Parameters	Normotension (MAP 65–80 mmHg)	Hypotension (MAP <65 mmHg)	*P*-value
HR (bpm)	97 (88–112)	96 (93–113)	0.9373
RR (mpm)	12 (10–14)	11 (10–15)	0.9572
SAP (mmHg)	105 ± 15	78 ± 12	0.0012
MAP (mmHg)	73 ± 5	56 ± 4	0.0027
DAP (mmHg)	60 ± 6	46 ± 3	0.0079
ETCO_2_ (mmHg)	40 (37–43)	39 (36–42)	0.6913
ET_ISO_ (mmHg)	1.4 (1.00–1.82)	2.0 (1.57–2.47)	0.0340
SpO_2_ (%)	98 (97–99)	97 (96–99)	0.9685

HR: heart rate; bpm: beats per minute; RR: respiratory rate; mpm: movements per minute; SAP: systolic arterial pressure; MAP: mean arterial pressure; DAP: diastolic arterial pressure; ETCO_2_: end-tidal carbon dioxide concentration; ET_ISO_: end-tidal isoflurane concentration; SpO_2_: pulse oxymetry. Mean ± SD; paired Student t-test (*P* < 0.05). Median (interquartile interval); Wilcoxon test (*P* < 0.05)

**Table 3:** Cardiac output (L/min) of 8 dogs assessed by thermodilution technique (TD) and by transesophageal echocardiography (TEE) during a mean arterial pressure of 65–80 mmHg (normotension) and <65 mmHg (hypotension)

	Normotension		Hypotension	
Dog	TD	TEE	TD	TEE
1	2.35	2.26	1.69	1.9
2	4.79	4.78	1.76	2.12
3	3.24	3.24	2.64	3.27
4	2.85	2.79	1.88	2.45
5	2.77	2.77	3.05	2.63
6	3.17	3.14	1.96	2.66
7	2.88	2.95	3.01	3.23
8	2.89	3.02	1.82	1.77
Mean ± SD	3.11 ± 0.72	3.11 ± 0.73	2.22 ± 0.57	2.50 ± 0.56

The ET_ISO_ was 1.4% (1.00–1.82%) during normotension and 2.0% (1.57–2.47%) during hypotension (Table 2). No other treatments but decreasing ET_ISO_ was necessary to normalise MAP after isoflurane-induced hypotension.

**Fig. 2:**
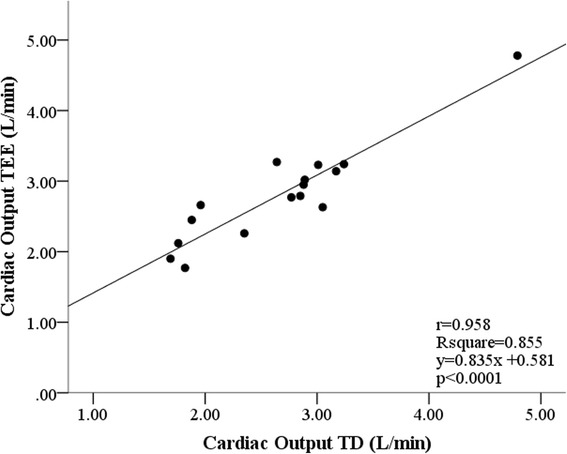
Graphical dispersion of cardiac output (CO) values obtained by transoesophageal echocardiography (TEE) and the thermodilution technique (TD) in anaesthetised dogs undergoing surgery

A strong positive correlation was found between CO_TEE_ and CO_TD_ (*r* = 0.925; *P* < 0.0001). The ICC between CO_TEE_ and CO_TD_ was 0.920 (*P* < 0.0001) for isolate measurements and 0.958 (*P* < 0.0001) for the mean of measurements. The linear regression analysis between CO_TEE_ and CO_TD_ was *r* ^2^ = 0.855 (*P* < 0.0001) and resulted in the following equation: CO_TEE_ = 0.581+ 0.835 × CO_TD_ (Fig. 2).

**Fig. 3:**
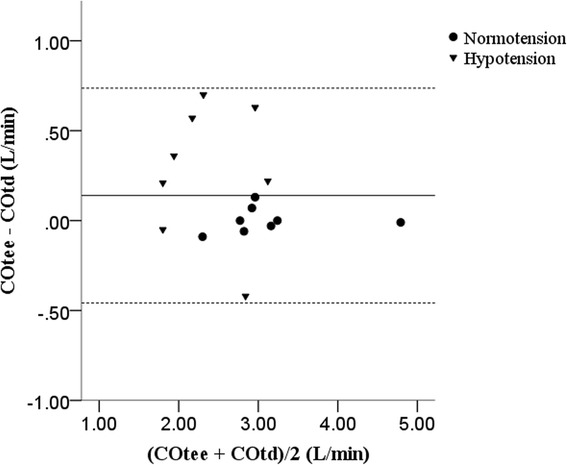
Bland-Altman analysis of cardiac output (CO) measured by transoesophageal echocardiography (TEE) and thermodilution technique (TD) in eight dogs with a mean arterial pressure of 65–80 mmHg (normotension) and <65 mmHg (hypotension) arterial pressure. All comparisons were within the thresholds of agreement (dotted line)

The agreement between CO_TEE_ and CO_TD_ showed a bias of 0.14 ± 0.29 L/min with a limit of agreement from −0.44 to 0.72 L/min (Fig. 3). A 12.32% error was found for the CO measured by both techniques.

**Fig. 4:**
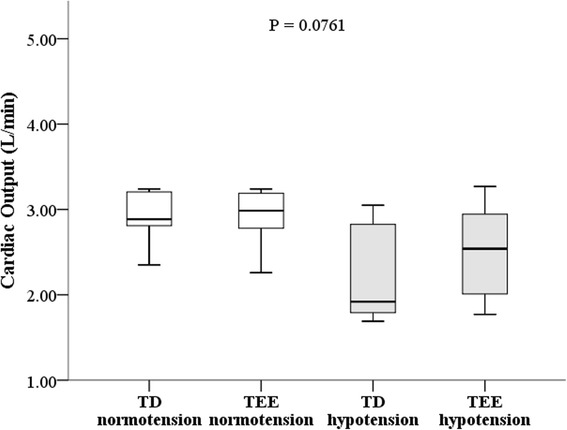
Cardiac output measured in anaesthetised dogs with an adequate (normotension) and low (hypotension) arterial pressure during surgery. The line within the box indicates the median of the observations. The top and low parts of the box indicate the first and the third quartile, respectively. There were no significant differences between the cardiac output measured by the thermodilution technique (TD) and the transoesophageal echocardiography (TEE) during arterial normotension (*P* = 0.9612) and hypotension (*P* = 0.0761), according to the results obtained with the Student paired t-test

There were no significant differences between CO_TEE_ and CO_TD_ during normotension (*P* = 0.9612) and hypotension (*P* = 0.0761) (Table 3; Fig. 4). A strong positive correlation was found between CO_TEE_ and CO_TD_ during normotension (*r* = 0.995; *P* < 0.0001) and hypotension (*r* = 0.78; *P* = 0.0223). The ICC between CO_TEE_ and CO_TD_ during normal arterial pressure was 0.995 (*P* < 0.0001) for isolate measurements and 0.998 (*P* < 0.0001) for the mean of the measurements. During arterial hypotension, the ICC between CO_TEE_ and CO_TD_ was 0.780 (*P* < 0.007) for isolate measurements and 0.876 (*P* < 0.007) for the mean of the measurements.

The linear regression analysis between CO_TEE_ and CO_TD_ resulted in a *r* ^2^ = 0.991 (*P* < 0.0001) and an equation of CO_TEE_ = 0.044 + 0.985 × CO_TD_ during normal blood pressure, and a *r* ^2^ = 0.609 (*P* = 0.022) and CO_TEE_ = 0.221 + 0.801 × CO_TD_ during hypotension.

The measurements bias for the condition of normotension and hypotension was 0.001 ± 0.07 L/min (limit of agreement: −0.13 to 0.14 L/min) and 0.27 ± 0.37 L/min (limit of agreement −0.46 to 1.01 L/min), respectively. A 2.55% error was found for the CO measured by both methods during normal arterial pressure and 15.26% during hypotension.

The coefficient of variation for the intraobserver and interobserver analysis for the CO_TEE_ measurements ranged from 0.47% to 7.77% and from 0.63% to 8.66%, respectively.

All dogs had ventricular arrhythmia during insertion of the pulmonary artery catheter that resolved spontaneously. With regard to the TEE, no complications were recorded.

## Discussion

The study showed that the TEE transgastric approach of the LVOT has a clinically acceptable correlation and agreement with the gold standard thermodilution technique, and that it can be used for measuring CO in dogs during surgery.

Invasive CO monitoring during surgery is infrequently performed in dogs using the pulmonary artery catheter [3] due to many factors, including associated complications such as arrhythmias, thromboembolism or pulmonary artery rupture [5], the availability of equipment and costs. The high incidence of cardiac arrhythmia observed in the present study indicates that the risk-benefit ratio of the right cardiac catheterisation should be considered according to the haemodynamic status of the patient. In contrast, the TEE was shown to be a less invasive technique with no cardiopulmonary complications and with an availability similar to that described in human patients for CO monitoring during surgery [7, 8].

Previous studies in veterinary patients have described the technique and the views obtained by the TEE [12, 13]. However, to the knowledge of the authors, this is the first clinical study to describe the application of the transgastric view of the LVOT for assessing CO in dogs during surgery. This echocardiographic window was obtained in all dogs of the study with an availability similar to that described for human patients [7, 8, 10].

The accuracy of the transthoracic echocardiography for measuring CO was demonstrated by other authors [14, 15], but the technique is often inappropriate for monitoring CO during surgery. TEE uses the same methodology as transthoracic echocardiography for calculating CO. Through this methodology, the CO is calculated by multiplying the SV with the HR [14]. To measure the stroke volume of the left ventricle, the cross-section area of the aorta is multiplied by the velocity-time integral aortic flow obtained by spectral Doppler [15]. By using the transgastric view of the LVOT, it was possible to obtain a parallel alignment between the volume sampled by the pulsate Doppler and the aortic blood flow and, therefore, to decrease bias when measuring the stroke volume [8].

A strong correlation was found between the techniques TEE and TD to obtain the CO, similar to that described in human patients [7, 8]. According to Bland & Altman [16], the correlation coefficient and regression analysis are not adequate statistical analyses for comparing two methods of measurement. The data analysis through the Bland-Altman technique is considered more adequate for assessing the agreement between the techniques, because it excludes the errors obtained from the use of the correlation coefficient, as it assesses the association of the measurements rather than the agreement between them. The agreement between techniques is considered satisfactory according to the Bland-Altman method, when the percentage error has a value lower than 30% [17]. In the present study, the error percentage between TEE and TD was 2.55% during normotension and 15.26% during hypotension, and there were no significant differences between CO calculated by TEE and TD in both arterial pressure conditions.

Coefficients of variation for intra- and interobserver measurement variability to the COTEE were lower than 10%, similar to that reported by other studies using transthoracic echocardiography [15, 17].

During the study, hypotension was induced in all animals during surgery and then reversed by reducing the isoflurane supply and/or administrating LR bolus. The decrease in arterial pressure was expected since isoflurane is an anaesthetic drug that reduces vascular resistance and that can be used as an inductor of hypotension in dogs undergoing experimental studies [18]. Despite an increase in HR expected during hypotension, this was not observed due to a decrease in the baroreflex induced by isoflurane and due to an increase in parasympathetic activity caused by fentanyl [19].

Some limitations of the study should be discussed. The TD technique can be relatively imprecise when measuring CO due to factors that can influence the accuracy of the measurement, such as dead space of the catheter, velocity of the administration and temperature of the solution [2]. However, this is still considered the gold standard for CO determination [3]. Despite the results of the present study having demonstrated that the TEE is an alternative technique to TD for measuring CO, in some situations, such as in surgeries of the oral, oesophageal or gastric areas, this technique cannot be applied.

## Conclusion

The results of the study indicated that the transgastric view of the LVOT acquired by transoesophageal echocardiography is a minimally invasive alternative to TD for monitoring CO in anaesthetised dogs during surgery. However, during hypotension, the CO obtained by TEE was less reliable, although still acceptable.

## Abbreviations

CO: Cardiac output
CO_TD_: Cardiac output measured by thermodilution technique
CO_TEE_: Cardiac output measured by transoesophageal echocardiography
DAP: Diastolic arterial pressure
ETCO_2_: End-tidal carbon dioxide concentration
ET_ISO_: End-tidal isoflurane concentration
HR: Heart rate
ICC: Intraclass correlation coefficient
LR: Lactated Ringer’s solution
LVOT: Left ventricular outflow tract
MAP: Mean arterial pressure
SAP: Systolic arterial pressure
SpO_2_: Oxyhaemoglobin saturation
TD: Thermodilution technique
TEE: Transoesophageal echocardiography
VTI: Velocity-time integral
VTI_aortic_: Velocity-time integral of aortic flow
